# AhR Promotes the Development of Non-small cell lung cancer by Inducing SLC7A11-dependent Antioxidant Function

**DOI:** 10.7150/jca.82066

**Published:** 2023-03-27

**Authors:** Yuanhao Peng, Lianlian Ouyang, Yangying Zhou, Weiwei Lai, Yuanbing Chen, Zuli Wang, Bokang Yan, Zewen Zhang, Yanling Zhou, Xintong Peng, Jielin Chen, Xin Peng, Desheng Xiao, Shuang Liu, Yongguang Tao, Wenliang Liu

**Affiliations:** 1 Department of Thoracic Surgery, Hunan Key Laboratory of Early Diagnosis and Precision Therapy in Lung Cancer, Second Xiangya Hospital, Central South University, Changsha, Hunan,410011, China; 2 NHC Key Laboratory of Carcinogenesis, Cancer Research Institute and School of Basic Medicine, Central South University, Changsha, Hunan, 410078, China; 3 Department of dermatology, Second Xiangya Hospital, Central South University, Changsha,410011, China; 4 Department of Oncology, Xiangya Hospital, Central South University, Changsha, Hunan, 410008, China; 5 Department of Neurosurgery, ​Xiangya Hospital, Central South University, Changsha, Hunan, 410008, China; 6 Center for Tissue Engineering and Stem Cell Research, Guizhou Medical University, Guizhou, 550025, China; 7 Department of Pathology, Zhuzhou Hospital Affiliated to Xiangya School of Medicine, Central South University, Zhuzhou, Hunan, 412007, China; 8 Department of Pathology, School of Basic Medicine and Xiangya Hospital, Central South University, Changsha, Hunan, 410008, China

**Keywords:** Receptors, aryl hydrocarbon, Ferroptosis, non-small-cell lung cancer, SLC7A11

## Abstract

**Objective:** Aryl hydrocarbon receptor (AhR) is a transcription factor. It is reported that AhR is associated with non-small cell lung cancer (NSCLC), but the mechanisms underlying this relationship remain unclear. Therefore, we investigated the role of AhR in NSCLC to elucidate the underlying mechanisms.

**Methods:** We collected clinical lung cancer samples and constructed *AhR* overexpression and knockdown cell lines to investigate the tumorigenicity of *AhR in vivo* and *in vitro*. Furthermore, we performed a ferroptosis induction experiment and chromatin immunoprecipitation experiment.

**Results:** AhR was highly expressed in NSCLC tissue. AhR knockdown cells showed ferroptosis related phenomenon. Furthermore, Chromatin immunoprecipitation confirmed the correlation between *AhR* and solute carrier family 7 member 11 (*SLC7A11*) and ferroptosis induction experiment confirmed that AhR affects ferroptosis via *SLC7A11*. Specifically, AhR regulates ferroptosis-related *SLC7A11*, which affects ferroptosis and promotes NSCLC progression.

**Conclusions:** AhR promoted NSCLC development and positively correlated with* SLC7A11*, affecting its actions. *AhR* bound to the promoter region of *SLC7A11* promotes NSCLC by activating *SLC7A11* expression, improving the oxidative sensitivity of cells, and inhibiting ferroptosis. Thus, AhR affects ferroptosis in NSCLC by regulating *SLC7A11*, providing foundational evidence for novel ferroptosis-related treatments.

## Introduction

Globally and in China, lung cancer is one of the most common malignancies with the greatest fatalities. More than 80% of lung cancers are non-small cell lung cancer (NSCLC). Furthermore, air pollution is becoming increasingly worse as China enters the late stage of industrialization. Thus, the incidence of NSCLC in China will likely continue to increase over the next 30 years. Therefore, understanding the pathogenesis of and how to prevent NSCLC is crucial 1, 2.

Aryl hydrocarbon receptor (AhR) is a member of the transcription factor family that requires ligand activation, and its classic high-affinity ligand, tetrachlorodibenzo-p-dioxin, is a common industrial pollutant 3. AhR is a multi-functional protein involved in growth, development, inflammation, tumors, the cell cycle, and cell proliferation, differentiation, and migration 4-6. Currently, controversy exists about AhR's role in cancer and cancer cells. AhR directly and indirectly regulates the expression of various genes, such as those for vital functional proteins (e.g., interleukin-6 and -21 [cytokines], vascular endothelial growth factor, epiregulin, and amphiregulin [growth factors], cyclin D, and matrix metalloproteinase [a matrix protein]) and transcription factors (e.g., Slug) 7. Therefore, the functions and mechanisms of AhR often vary among tissues, cells, and their molecular microenvironments 8-10.

The latest research shows that benzopyrene, found in cigarette smoke and air pollutants, can induce programmed death-ligand 1 expression in lung epithelial cells through AhR; thus, it escapes the body's adaptive immunity, promoting tumor occurrence 11. Our previous study found that benzopyrene activates AhR nuclear transport, leading to the malignant transformation of NSCLC 9. Others have also reported that AhR has an important role in tumor development 7, 12 and that inhibiting its signaling pathway with AhR antagonists significantly inhibited the malignant evolution and invasion of tumors 13-15. Our previous research also found that AhR independently activates downstream target genes 16, AhR pathway activation has been associated with stem-like peculiarities and radiation resistance in tumor cells 17 and can be stabilized by de-ubiquitination 18. Therefore, downstream molecules are recruited for various roles after exogenous factors activate AhR, which may affect the expression of ferroptosis-related genes, such as *SLC7A11*, and thus the ferroptosis process.

Cell death occurs at the end of a cell's life, with essential roles in the body's survival and development. Ferroptosis is a new form of cell death, differing from apoptosis, autophagy, and necrosis. This type of cell death primarily depends on iron metabolism and is characterized by the accumulation of reactive oxygen species (ROS) 19, 20. Intracellular signaling pathways closely regulate ferroptosis, including the iron homeostasis regulatory, RAS, and cystine transport pathways 21. In addition, cystine transport plays an essential role in regulating ferroptosis 22; cystine replaces x_c_^-^ intracellular glutamate via the cystine-glutamate antiporter system, and excessive lipid ROS causes ferroptosis 23. Solute carrier family 7 member 11 (*SLC7A11*) is a key component of system x_c_^-^, and reports indicate that oxidative and metabolic stress can induce *SLC7A11* expression 24, 25. Furthermore, the activity and stability of *SLC7A11* regulate the cells' antioxidant capacity 26 and then affect ferroptosis 27. Thus, *SLC7A11* is a key regulatory molecule for ferroptosis.

To date, the mechanisms underlying the associations among AhR, NSCLC, and ferroptosis remain unclear. Therefore, this study investigated the potential effects of AhR on ferroptosis and NSCLC to elucidate the underlying mechanisms.

## Materials and Methods

### Cell culture, plasmids, short hairpin RNAs (shRNAs), chemicals, and antibodies

We used PC9, SPC-A-1, and A549 cell lines and followed previously described maintenance methods 28, 29. The primary antibodies were β-actin (Cat# A5441; Sigma-Aldrich, St. Louis, MO, USA; 1:10000), AhR (Cat# sc-133088; Santa Cruz Biotechnology, Santa Cruz, CA, USA; 1:1000), and SLC7A11 (Cat# 26864-1-AP; Proteintech, Wuhan, China; 1:1000).

We used previously published shRNA sequences 17. Plasmid transfection was performed with Lipofectamine® 3000 following the manufacturer's instructions; colonies with stable expression were screened using puromycin (1.5 μg/mL). Vigene Biosciences designed and established the AhR overexpression plasmid (Vigene Biosciences, Shandong, China).

AhR agonist V, VAF347 was purchased from Calbiochem (Cat# 182690; Calbiochem/EMD Chemicals, San Diego, CA, USA), and Erastin (Cat# S7242) and Ferrostatin-1 (Cat# S7243) were purchased from Selleck Chemicals (Houston, TX, USA).

### Cell proliferation, migration and invasion, and plate colony-formation assays

We followed the previously described procedures by SY et al. and JY et al. 30, 31.

### Chromatin immunoprecipitation (ChIP) assays

The ChIP assays were performed as previously described by JY et al. 31. ChIP DNA was detected using an ABI 7500 real-time polymerase chain reaction (PCR) instrument (Applied Biosystems, Foster City, CA, USA) using SYBR Green (Bio-Rad, Hercules, CA, USA). We used the anti-AhR antibody (Cat# sc-133088; Santa Cruz Biotechnology; 1:500).

### Western blot analysis

We followed previously described instructions for the Western blot analysis 31. We used mouse monoclonal anti-β-actin (Cat# A5441, Sigma-Aldrich, St. Louis, MO, USA; 1:10000), mouse monoclonal anti-AhR (Cat# sc-133088, Santa Cruz Biotechnology; 1:1000), and rabbit polyclonal anti-SLC7A11 (Cat# 26864-1-AP, Proteintech; 1:1000) for protein detection.

### Total and lipid ROS and intracellular iron measurements

We followed previously described procedures for these measurements 23.

### Flow cytometry

Cells were stained with antibodies or fluorescent dyes following standard protocols (D3861-BODIPY™ 581/591 C11; Thermo Fisher Scientific, Waltham, MA, USA) before being loaded for flow cytometry. Fluorescein-green and Texas-red were used to detect the labeled cells. The analysis was performed using FlowJo software (FloJo LLC., Ashland, OR, USA).

### Reverse transcription-quantitative real-time PCR

We followed previously described procedures 23.

### Histology and immunohistochemistry (IHC)

The Pathology Department of Xiangya Hospital in China provided the lung cancer samples. Details of the immunohistochemical analysis of paraffin sections of the lung cancer tissue have already been published 23. Two pathologists at Xiangya Hospital in Changsha, China, performed the differential quantifications.

### Nude mice experiment

Four-week-old female severe combined immune-deficiency mice were purchased from Hunan SJA Laboratory Animal Co., Ltd. (Hunan, China). The Animal Protection and Use Agency Committee of Xiangya Medical College of Central South University approved all animal experiment protocols, which complied with the legal authorization and federal animal protection and conservation guidelines.

Each mouse was subcutaneously injected with AhR overexpression or knockdown cells (1 × 10^6^ cells/animal). The control mice were injected with the same concentration of cells transfected with a control plasmid. The tumor volume and body weight were measured every three days until day 31.

### Statistical analyses

The experiments were repeated at least three times. The results are presented as means ± standard deviations, as indicated. All statistical analyses were performed using Prism GraphPad Software (version 8.0, GraphPad Software, San Diego, CA, USA). Student's t-tests were used to compare two groups, and an analysis of variance was used to compare three or more groups. P-values of <0.05 were considered statistically significant.

## Results

### AhR is upregulated in NSCLC at the protein level

**Figure 1:**
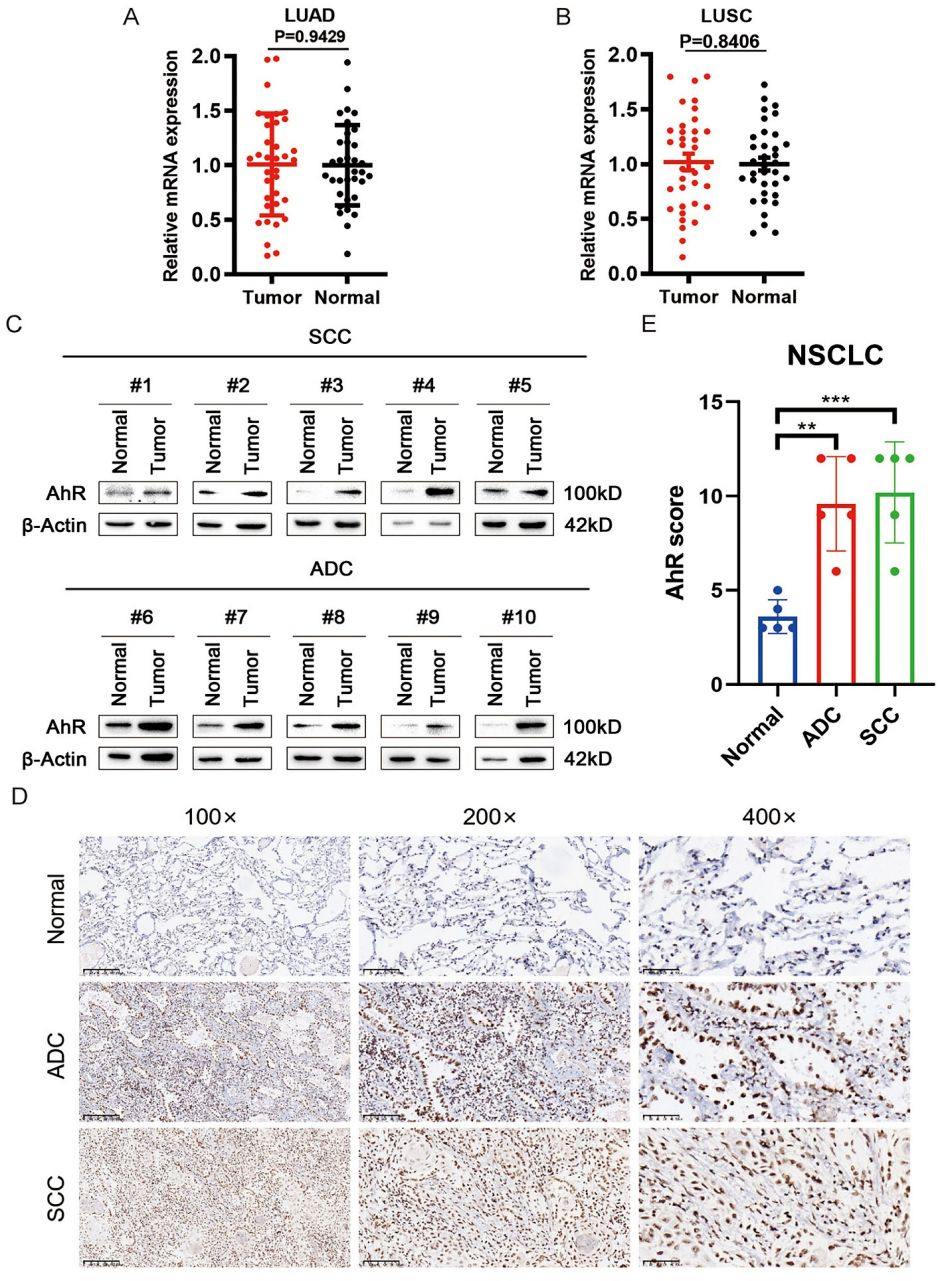
AhR is upregulated at the protein level in non-small cell lung cancer (NSCLC). Clinical Samples analysis of aryl hydrocarbon receptor (*AhR*) mRNA expression in A) Lung adenocarcinoma (LUAD) (Normal, n = 35; Tumor, n = 35) and B) Lung squamous cell carcinoma (LUSC) (Normal, n = 35; Tumor, n = 35). C) AhR protein expression is higher in lung cancer (adenocarcinoma [ADC] and squamous cell carcinoma [SCC]) tissue than in paired adjacent normal lung tissue (n = 10; western blot). D) Immunohistochemistry (IHC) analyses showed increased AhR expression levels in lung cancer tissues (100× magnification, scale bar = 200 μm; 200× magnification, scale bar = 100 μm; 400× magnification, scale bar = 50 μm). E) IHC scores showing AhR expression levels in lung cancer versus normal tissues (Normal: n = 8; SCC: n = 10; ADC: n = 10). Data are presented as means ± standard deviations; **p <0.01, ***p <0.01.

To clarify the role of AhR in NSCLC, we extracted mRNA from clinical non-small cell lung cancer samples for analysis, finding that the *AhR* mRNA level did not differ between lung adenocarcinoma (ADC; Fig. 1A) and squamous cell carcinoma (SCC; Fig. 1B) tissues and adjacent tissues. Subsequently, we evaluated AhR protein expression in ten NSCLC and adjacent normal tissue pairs by western blot (Fig. 1C), which showed upregulated AhR protein expression in both ADC and SCC tissues. This result suggested that *AhR* regulation occurred at the post-transcriptional level.

Simultaneously, we performed IHC analyses on NSCLC and adjacent normal tissue samples to confirm the AhR expression level. AhR expression was significantly higher in SCC and ADC tissues than in the normal adjacent tissue (Fig. 1D). Additionally, the AhR IHC scores in the SCC and ADC tissues were higher than those in adjacent normal tissues (Fig. 1E). These results suggested that AhR expression may increase in human NSCLC, and AhR may play a vital biological role.

### AhR knockdown inhibits NSCLC progression *in vitro* and *in vivo*

**Figure 2:**
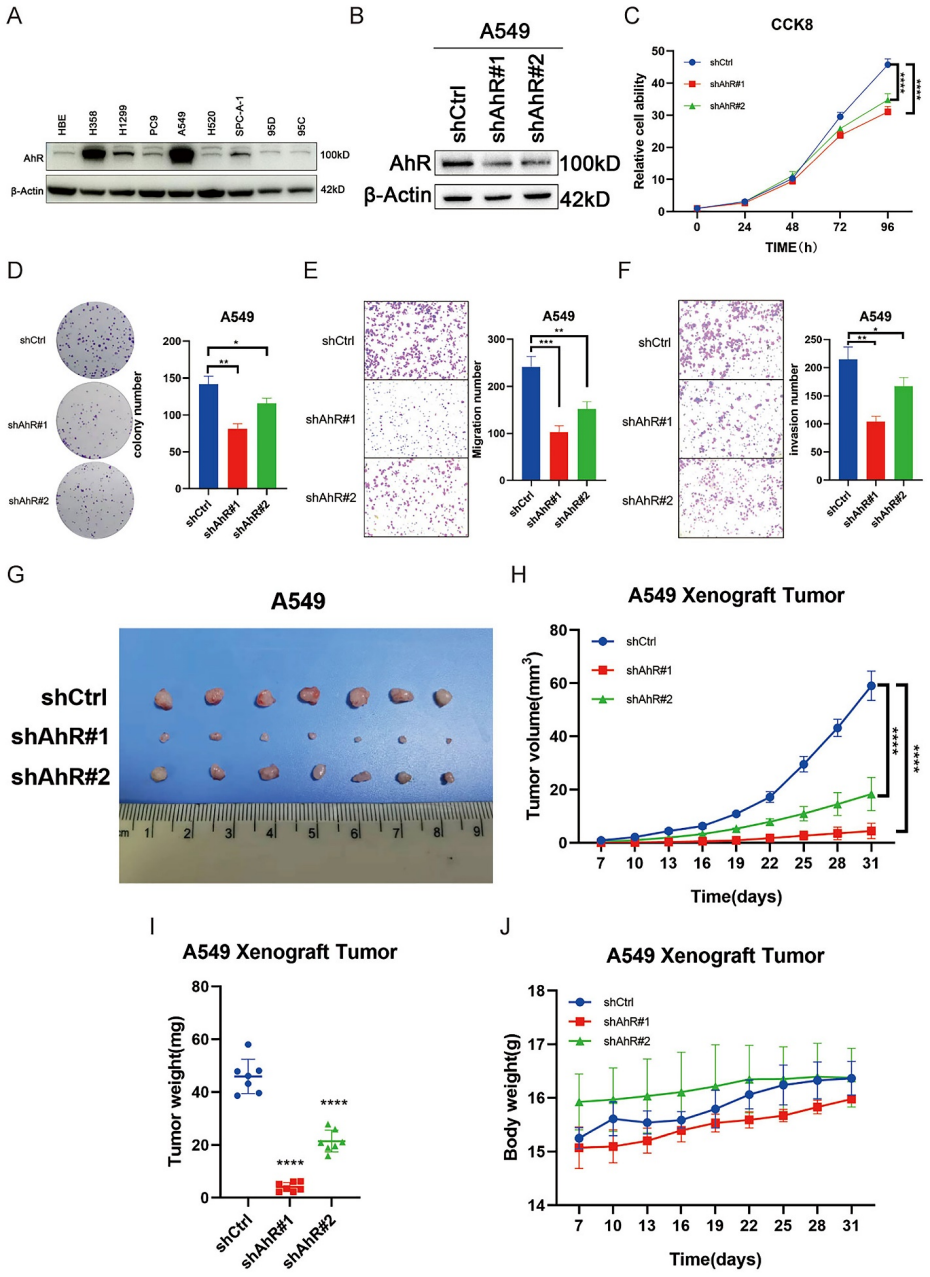
Aryl hydrocarbon receptor (*AhR*) knockdown inhibits NSCLC progression *in vitro* and *in vivo*. A) AhR protein expression levels in lung cancer cell lines. B) AhR protein expression after *AhR* knockdown in A549 cells (western blot). C) Cell viability in A549 cells with stable* AhR* knockdown (n = 5; MTS assay). Data are shown as means ± standard deviations (SDs); ****p <0.0001. D) Colony formation abilities of A549 cells with stable* AhR* knockdown (n = 3; colony formation assay in plates). Data are presented as means ± SDs; **p <0.01. Representative images of E) *AhR*-knockdown A549 cell migration (n = 3) and F) *AhR*-knockdown A549 cell invasion (n = 3). G-J) A xenograft nude mouse model was established using *AhR*-knockdown A549 or control cells, and then the tumor volume (G, H), weight (I) and mouse body weight (J) were measured over time.

We measured AhR protein expression levels in different lung cancer cell lines (Fig. 2A). We found low AhR levels in the PC9 and SPC-A-1 cells and an increased level in the A549 cells.

Next, we stably knocked down *AhR* in the A549 cell line using four different AhR-targeted shRNAs; shAhR #1 and shAhR #2 had relatively high knockdown efficiencies (Fig. 2B). Therefore, all experiments were performed with these two shRNAs, unless otherwise noted. The *AhR*-knockdown cells grow more slowly than the control cells (Fig. 2C) and had significantly inhibited colony-forming abilities (Fig. 2D). Furthermore, the *AhR*-knockdown cells had lower invasion and migration capacities than the carrier cells (Fig. 2E, F).

Finally, we injected A549 cells into nude mice to investigate tumor formation *in vivo*. *AhR* knockdown significantly reduced the tumor size, volume, and weight compared to the control mice (Fig. 2G-I), but body weight did not differ between the two groups (Fig. 2J). These results indicated that *AhR* promotes non-small cell lung cancer development and thus has carcinogenic properties.

### AhR overexpression promotes NSCLC progression

**Figure 3:**
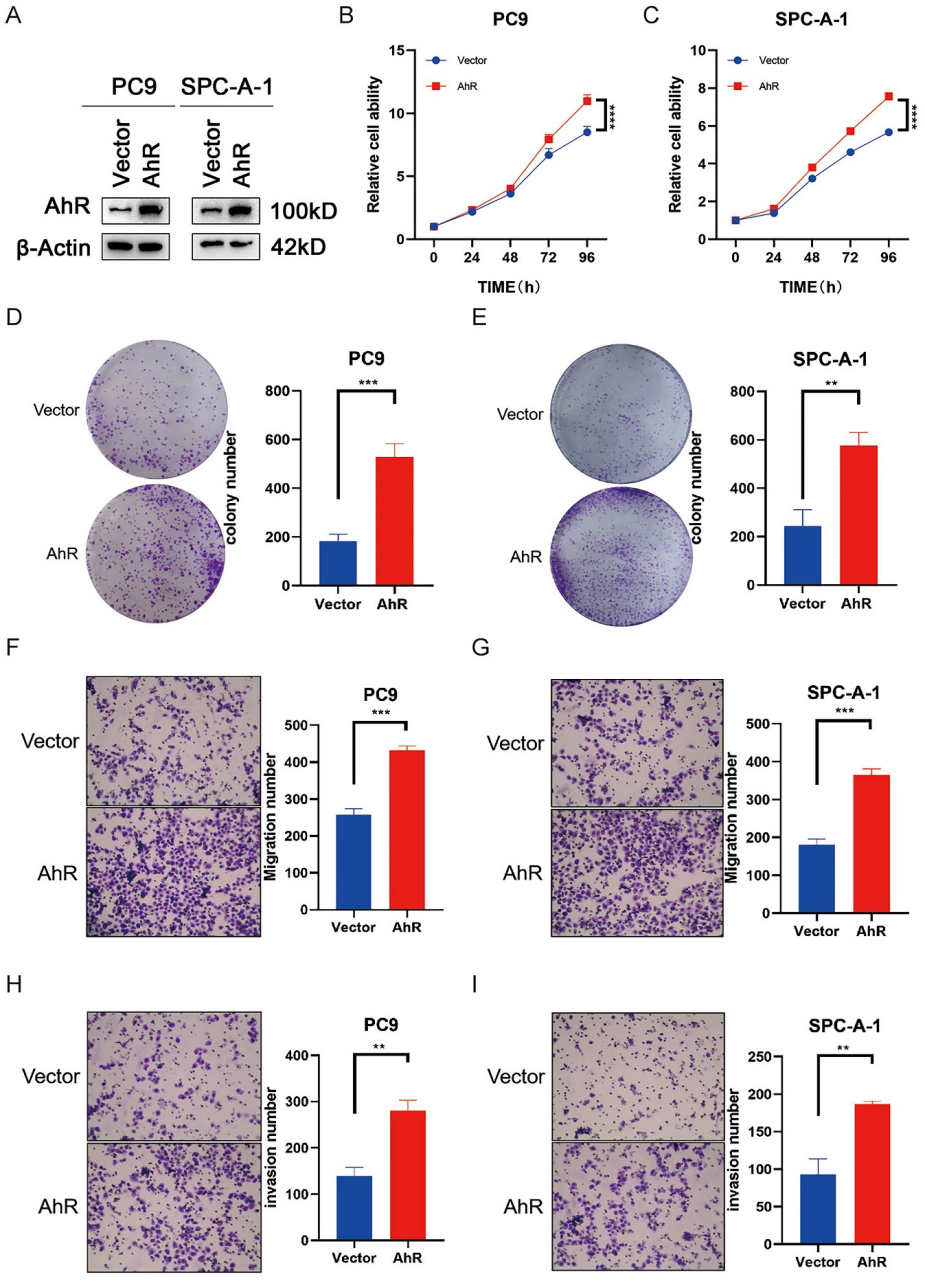
Aryl hydrocarbon receptor (*AhR*) overexpression promotes NSCLC progression. A) AhR protein levels in PC9 and SPC-A-1 cells overexpressing *AhR* (western blot). B, C) Cell viability in PC9 and SPC-A-1 cells with stably overexpressed *AhR* (n = 5; MTS assay). Data are presented as means ± standard deviations (SDs); ****p <0.0001. D, E) Colony formation abilities of PC9 and SPC-A-1 cells with stably overexpressed *AhR* (n = 3; colony formation assay in plates). Data are presented as means ± SDs; **p <0.01. Representative image of F, G) *AhR*-overexpressing PC9 and SPC-A-1 cell migration (n = 3) and H, I) *AhR*-overexpressing PC9 and SPC-A-1 cell invasion (n = 3).

To clarify the physiological effects of AhR in NSCLC, we overexpressed AhR in PC9 and SPC-A-1 cells. Then, we assessed the overexpression efficiencies by western blot (Fig. 3A). *AhR* overexpression promoted PC9 and SPC-A-1 cell growth *in vitro* (Fig. 3B, C) and significantly improved their colony-forming abilities (Fig. 3D, E). In addition, the cell lines overexpressing *AhR* had stronger invasion and migration capabilities than the carrier cells (Fig. 3F-I). These results confirmed that *AhR* overexpression is related to cell growth, colony formation and cell migration and has carcinogenic influences.

### AhR regulates ferroptosis through *SLC7A11*

**Figure 4:**
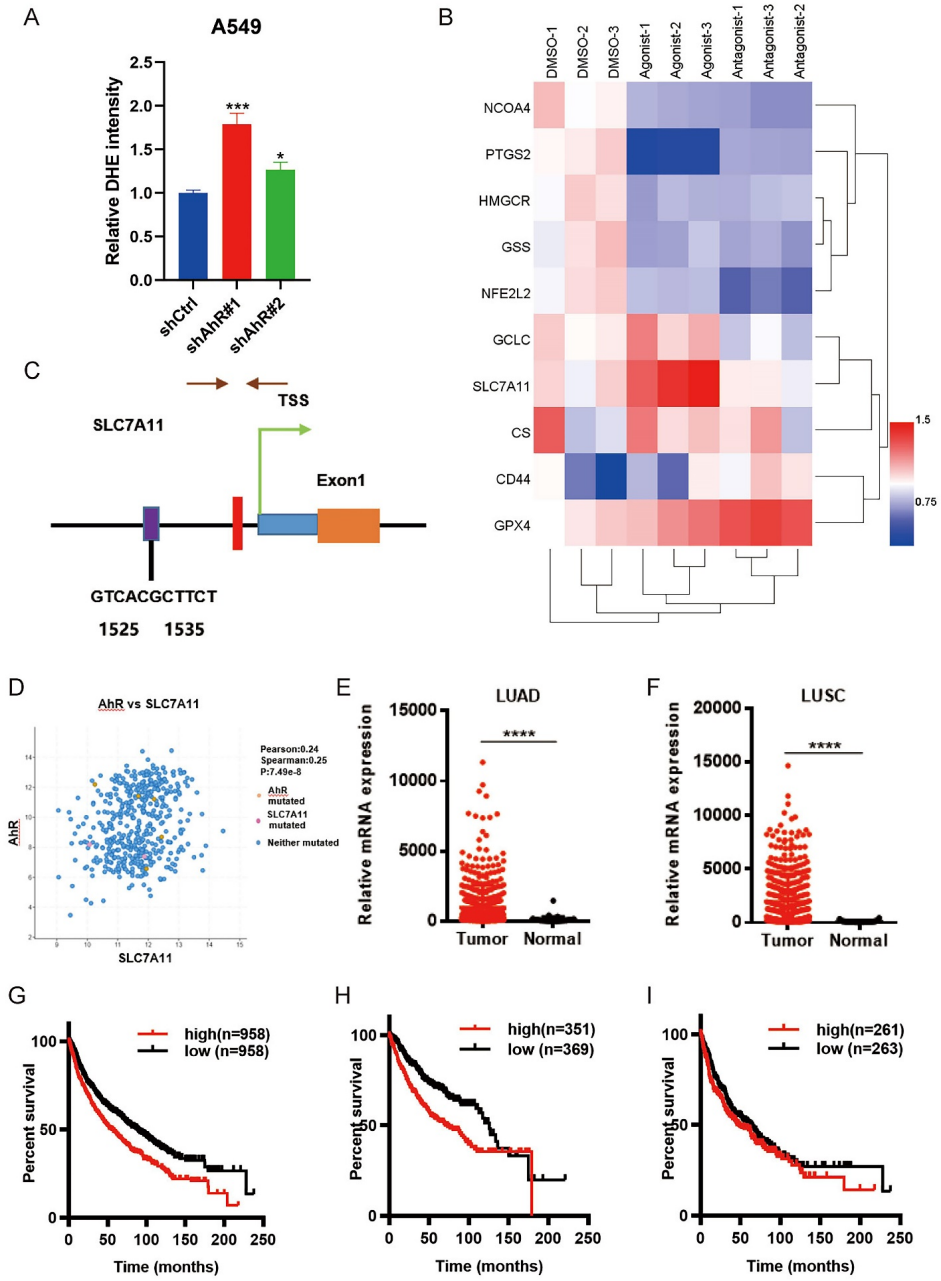
Aryl hydrocarbon receptor (*AhR*) regulates ferroptosis via solute carrier family 7 member 11 (*SLC7A11*). A) The total ROS level was higher in *AhR*-knockdown A549 cells than in A549 cells without knockdown. B) *AhR* binding site predictions in the *SLC7A11* promoter region using a University of California Santa Cruz Genome Browser database analysis and Algorithmics and Genetics Group (i.e., ALGGEN) website. C) Bioinformatics analysis identified a positive correlation between *AhR* and *SLC7A11*. D) *SLC7A11* expression in A549 cells was significantly upregulated after treatment with an *AhR* agonist; expression did not change after treatment with an AhR agonist. E, F) The Cancer Genome Atlas database analysis showed that *SLC7A11* expression in LUAD and LUSC is higher than that in adjacent tissues. G, I) High *SLC7A11* expression is unfavorable for survival and prognosis.

When we knock down *AhR*, we found that there was a phenomenon related to ferroptosis in cells, for example, the total ROS level of A549 cells knocked down *AhR* would increas (Fig. 4A), suggesting that AhR may be related to ferroptosis.

Therefore, we performed a preliminarily ferroptosis-related gene expression analysis in lung ADC cells, finding Significant differential *SLC7A11* expression after treating the cells with an *AhR* agonist and antagonist (Fig. 4B), suggesting that AhR may be related to SLC7A11.Furthermore, in order to explore the relationship between *AhR* and *SLC7A11*,we used the University of California Santa Cruz Genome Browser database and bioinformatics analyses showed that there might be *AhR* binding sites in the promoter region of *SLC7A11* (Fig. 4C). We also identified a positive correlation between *AhR* and *SLC7A11* (Fig. 4D). The Cancer Genome Atlas database analysis also showed higher *SLC7A11* expression in ADC and SCC tissue than in adjacent tissues (Fig. 4E, F). Furthermore, high *SLC7A11* expression was unfavorable for survival prognoses (Fig. 4G, I). Together, these results indicated that *AhR* might inhibit tumor cell ferroptosis by regulating *SLC7A11* expression.

### *SLC7A11* positively correlates with *AhR* in NSCLC

**Figure 5:**
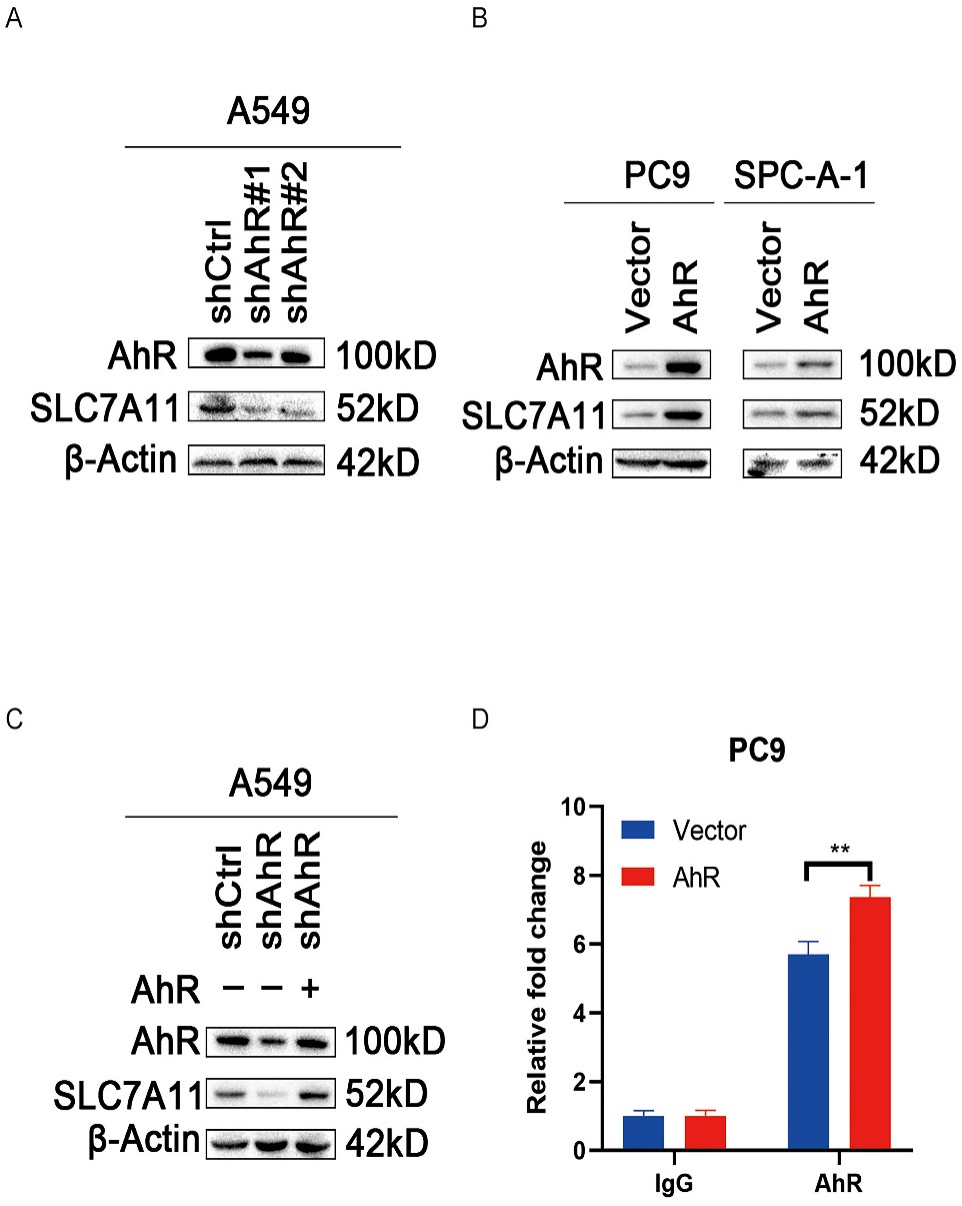
Solute carrier family 7 member 11 (*SLC7A11*) positively correlates with aryl hydrocarbon receptor (*AhR*) in non-small cell lung cancer (NSCLC). A) AhR and SLC7A11 protein levels in *AhR*-knockdown A549 cells (western blot). B) AhR and SLC7A11 protein levels in *AhR*-overexpressing PC9 and SPC-A-1 cells (western blot). C) AhR and SLC7A11 in *AhR*-knockdown A549 cells transfected into an *AhR*-overexpression plasmid (western blot). D) *AhR* in the promoter region of *SLC7A11* using two primer sites (chromatin immunoprecipitation assay).

Owing to previous analysis and experiments, we hypothesized a positive correlation between *AhR* and *SLC7A11*. To clarify this relationship, we detected the SLC7A11 protein level (by immunoblot) in lung cancer cell lines after overexpressing and knocking down *AhR*. The SLC7A11 protein level increased in the *AhR* overexpression cells (Fig. 5A) and decreased in the *AhR* knockdown cells (Fig. 5B). For further clarification, we transfected the *AhR* expression plasmid into the *AhR* knockdown A549 cell line, and then detected the AhR and SLC7A11 protein levels by western blot, which confirmed their positive relationship (Fig. 5C).

We also performed a ChIP analysis to detect *AhR* in the promoter region of *SLC7A11*. In PC9 cells, *AhR* was recruited into the *SLC7A11* promoter. Furthermore, *Ahr* recruitment into the *SLC7A11* promoter region was greater in PC9 cells overexpressing *AhR* than in the control cells (Fig. 5D).

### *AhR* overexpression inhibits ferroptosis in NSCLC

**Figure 6:**
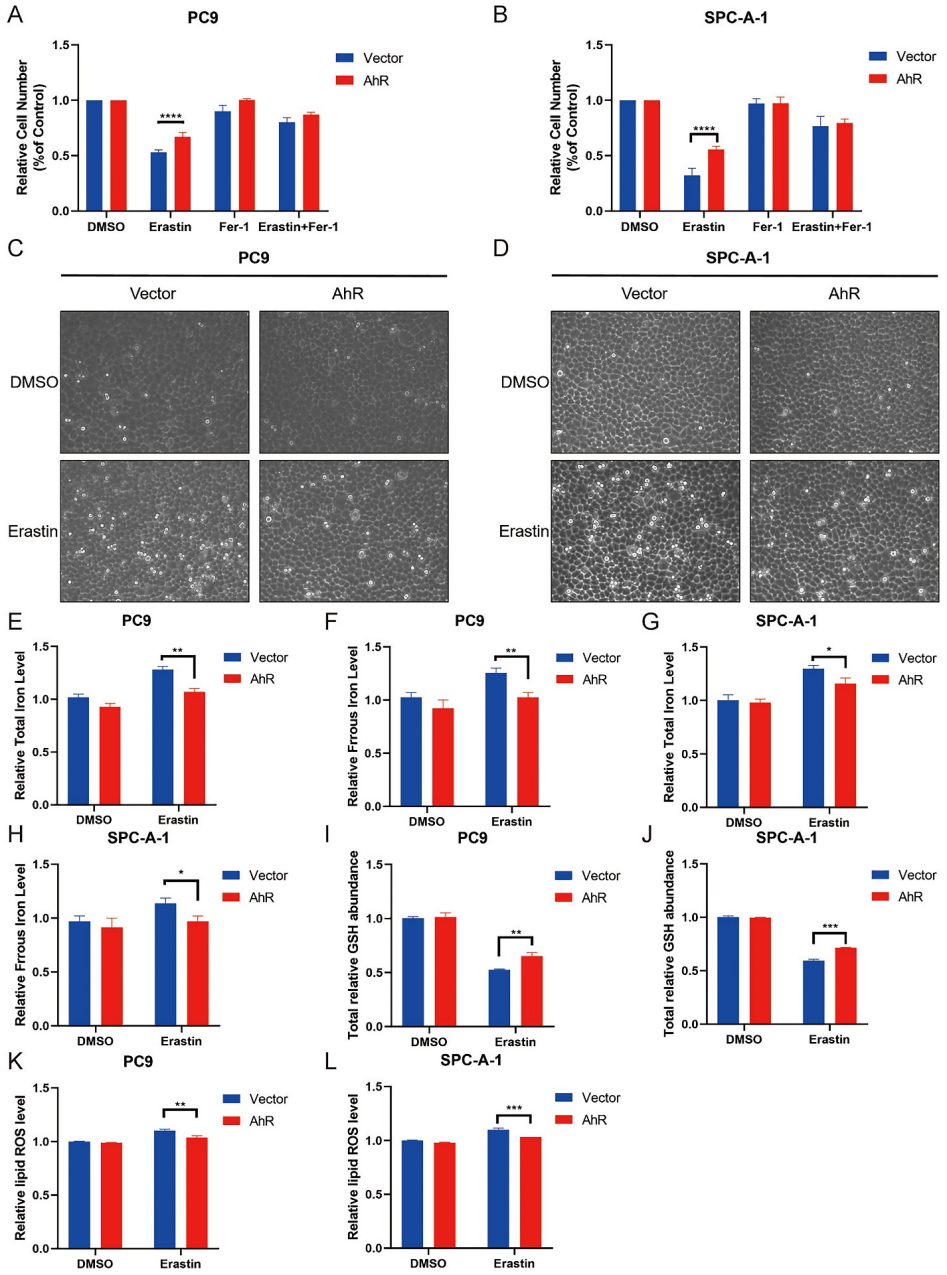
Aryl hydrocarbon receptor (*AhR*) overexpression inhibits NSCLC cell ferroptosis. A, B) PC9 (A) and SPC-A-1 (B) cell responses to Erastin (5 μM) ± Ferrostatin (1 μM) with or without *AhR* overexpression. C, D) A representative image showing PC9 (A) and SPC-A-1 (B) cell responses to Erastin (5 μM) ± Ferrostatin (1 μM) with or without *AhR* overexpression. E, F) Total iron (E) and ferrous iron (F) levels in PC9 cells with or without *AhR* overexpression. G, H) Total iron (G) and ferrous iron (H) levels in SPC-A-1 cells with or without *AhR* overexpression. I, J) The total relative glutathione (GSH) levels in PC9 (I) and SPC-A-1 (J) cells with or without *AhR* overexpression. K, L) The lipid reactive oxygen species (ROS) level was measured by C11-BODIPY staining coupled to flow cytometry in Erastin-treated PC9 (K) and SPC-A-1 (L) cells after *AhR* overexpression. *p <0.05, **p <0.01, ***p <0.001.

*SLC7A11* is a key molecule regulating ferroptosis, and we found that *AhR* positively correlated with *SLC7A11*, prompting us to explore the role of *AhR* in ferroptosis. First, we treated PC9 and SPC-A-1 cell lines overexpressing *AhR* with Erastin, a *SLC7A11* inhibitor, to evaluate cell growth inhibition, the drug concentration followed previously described instructions 28, 29. *Ahr* overexpression decreased the growth inhibition of Erastin-treated PC9 and SPC-A-1 cell lines (Fig. 6A, B). Morphological observations of control cells (PC9 cells treated with DMSO) versus Erastin-treated PC9 and SPC-A-1 cells overexpressing *AhR* supported this result (Fig. 6C, D). These results suggested that *AhR* inhibits Erastin-induced cancer cell death.

Iron, glutathione (GSH), and lipid ROS concentrations in cells are surrogate markers of ferroptosis 21, 32. We observed that PC9 and SPC-A-1 cells treated with Erastin had a higher concentration of iron than the control group, but *AhR* overexpression inhibited this increase (ferrous ions and total iron concentrations; Fig. 6E-H). Furthermore, Erastin treatment decreased the total GSH level, but *AhR* overexpression inhibited this reduction (Fig. 6I, J). Finally, Erastin treatment increased the lipid ROS level, but *AhR* overexpression inhibited this increase (Fig. 6K, L). These findings indicated that *AhR* overexpression inhibits Erastin-induced cancer cell ferroptosis.

### AhR knockdown promotes ferroptosis in NSCLC

**Figure 7:**
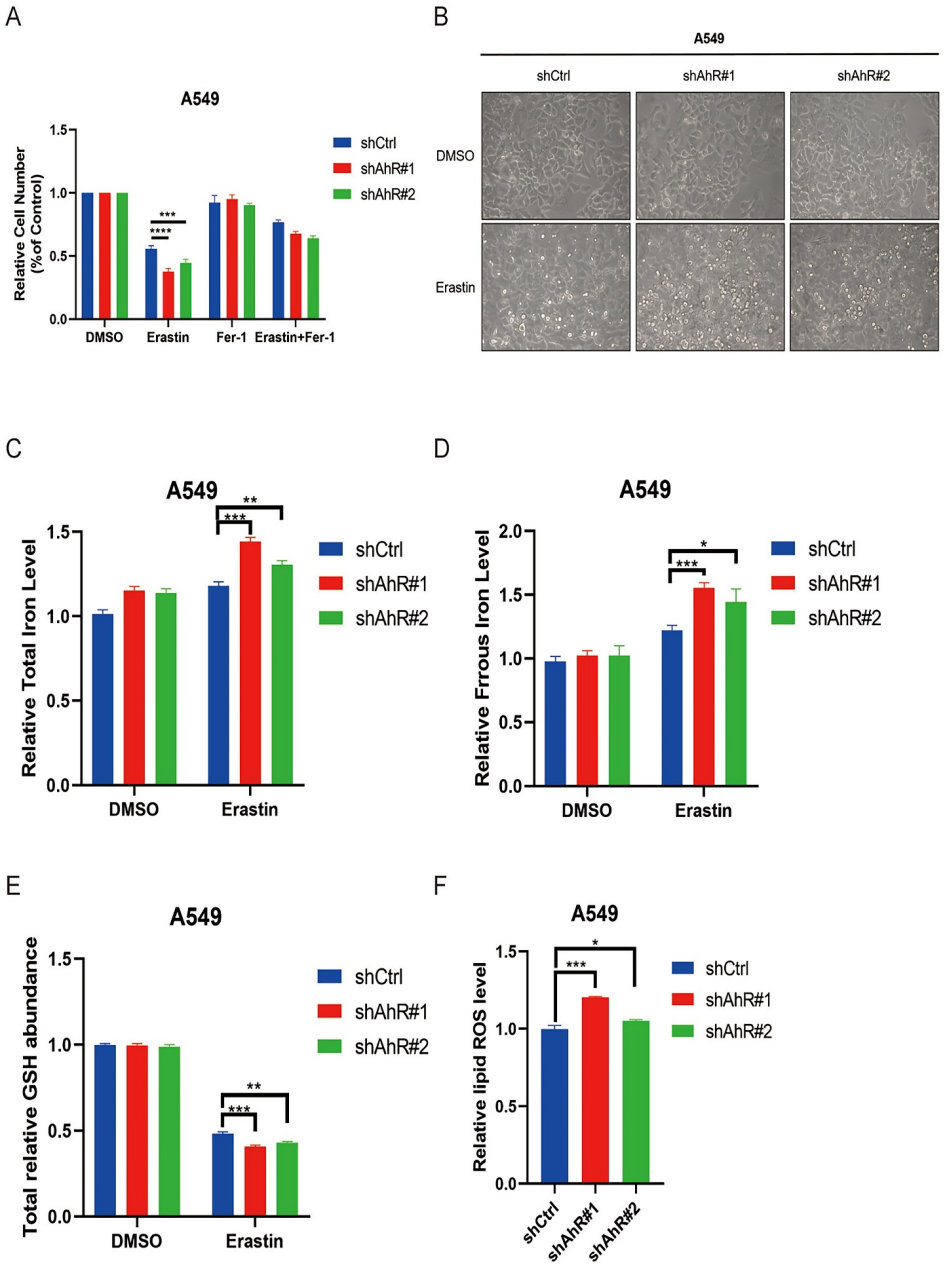
Aryl hydrocarbon receptor (*AhR*) knockdown promotes NSCLC cell ferroptosis. A) A549 cell responses to Erastin (5 μM) ± Ferrostatin (1 μM) with or without *AhR* knockdown. B) A representative image showing A549 cell responses to Erastin (5μM) ± Ferrostatin (1 μM) with or without *AhR* knockdown. C) The total iron levels in A549 cells with or without *AhR* knockdown. D) The ferrous iron levels in A549 cells with or without *AhR* knockdown. E) The total relative glutathione (GSH) levels in A549 cells with or without *AhR* knockdown. F) The lipid reactive oxygen species (ROS) level was measured by C11-BODIPY staining coupled to flow cytometry in Erastin-treated A549 cells after *AhR* knockdown. *p <0.05, **p <0.01, ***p <0.001.

To further clarify the role of *AhR* in ferroptosis in NSCLC, we tested the effects of Erastin on growth inhibition of *AhR*-knockdown A549 cells treated with Erastin. Knocking down *AhR* enhanced the Erastin-induced inhibitory effect on A549 cell growth (Fig. 7A); the morphological observation aligned with this finding (Fig. 7B).

We also measured the total iron and ferrous concentrations in A549 cells after knocking down *AhR*, which increased compared to cells without *AhR* knockdown (Fig. 7C, D). Furthermore, the total GSH level was obviously lower in *AhR* knockdown cells than in those without knockdown (Fig. 7E). Finally, the lipid ROS level of A549 cells after *AhR* knockdown significantly increased after Erastin treatment compared to the DMSO group (Fig. 7F). Therefore, *AhR* knockdown promotes ferroptosis of Erastin-induced cancer cells.

**Figure 8:**
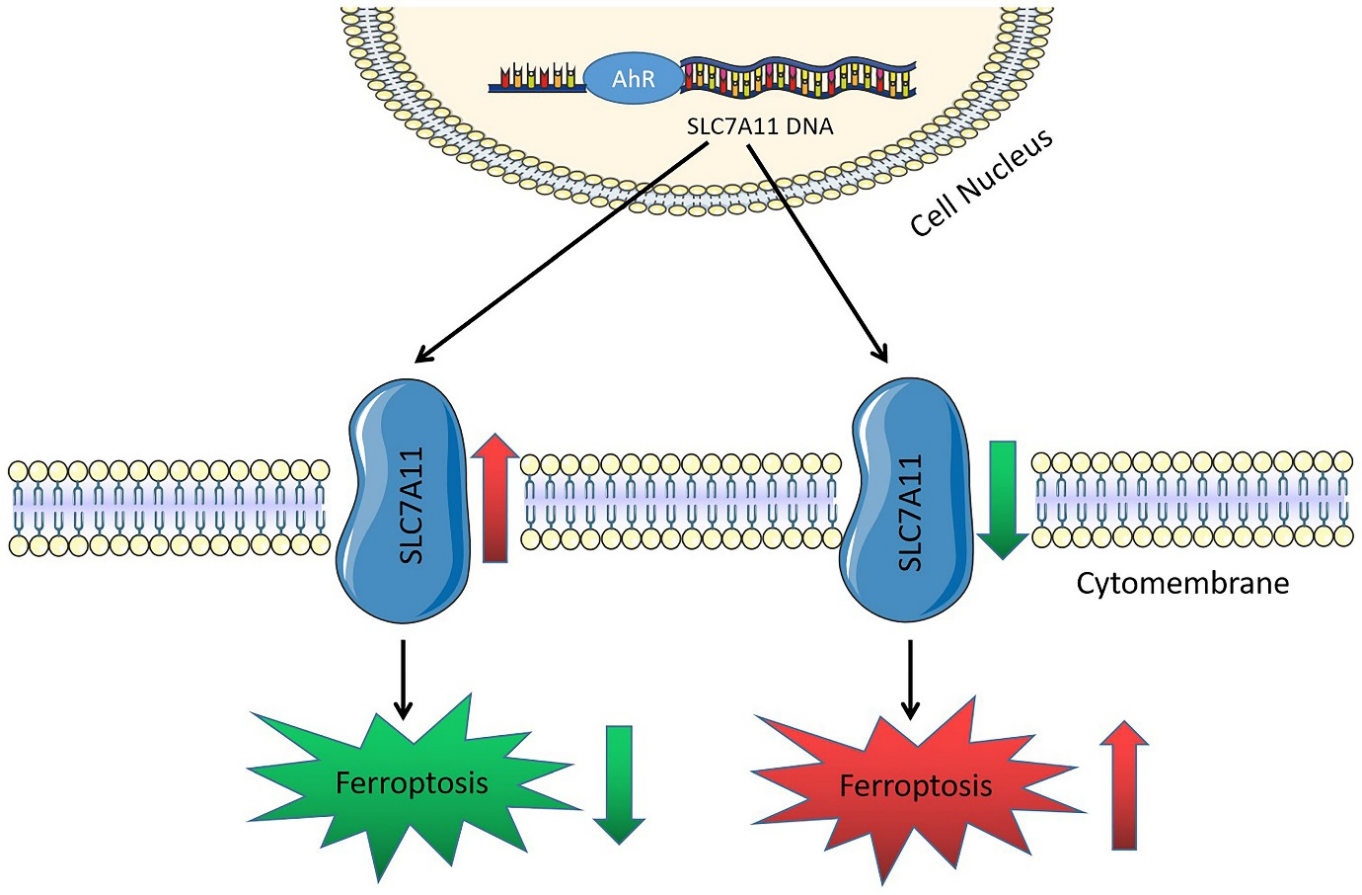
Schematic working model of solute carrier family 7 member 11 (*SLC7A11*) regulation by aryl hydrocarbon receptor (AhR). AhR is a transcription factor. The *SLC7A11* promoter region increases its expression by recruiting AhR, inhibiting ferroptosis, and promoting cancer development.

Overall, *AhR* may regulate iron, GSH, and lipid ROS levels in lung cancer cells by promoting *SLC7A11* transcription, thereby inhibiting lung cancer cell ferroptosis and promoting non-small cell lung cancer development (Fig. 8).

## Discussions

This study provides new evidence that *AhR* plays an important role in non-small cell lung cancer development. For the first time, we demonstrate that *AhR*, a ferroptosis inhibitor, influences carcinogenesis by promoting *SLC7A11*, an antioxidant system-related gene. Ferroptosis is a type of programmed cell death and a promising new target for treating tumors. Studies have reported strong relationships between ferroptosis and tumor progression 32-36, for example, ferroptosis-mediated radiation therapy for tumors 32 and tumor killing by immune cells 37. Additionally, reports indicate that ferroptosis-related genes have vital roles in pancreatic cancer 38-40, suggesting that ferroptosis may be a novel way to inhibit cancer development.

Transcription factor AhR is a protein with several functions 41-43, including cell growth, development, proliferation, and tumor differentiation 7, 44. Its functions and mechanisms often vary based on tissue and cell type and their molecular microenvironment 45-49. AhR is highly expressed in NSCLC, but there is no change in RNA level, the previous research results of our group have confirmed that this is based on the effect of de-ubiquitination 18. Therefore, AhR is important for tumorigenesis, but how it affects tumor progression remains unclear. Elucidating these mechanisms will allow the identification of new targets for treating AhR-positive cancer. Similarly, *AhR* expression varies among NSCLCs, and the mechanism underlying the abnormal *AhR* expression remains unclear. Additionally, the results are inconsistent, with several reports indicating *AhR* downregulation in lung cancer 50, but others reported *AhR* overexpressed 51, 52, consistent with our findings. Furthermore, some reports indicating *AhR* overexpression promoted NSCLC development.

Ferroptosis is characterized by an overwhelming, iron-dependent accumulation of lethal lipid ROS 53-55. We demonstrated that *AhR* overexpression decreased the lipid ROS and iron concentrations and increased the total GSH concentration in human lung ADC cell lines, supporting *AhR* inhibition in ferroptosis. Ferroptosis is also characterized by an increase in lipid peroxide, which may be caused by compound-mediated inhibition of GSH peroxidase 4 (GPX4) or indirectly targeting GPX4 through GSH consumption. Additionally, system x_c_^-^ is an important antioxidant system in cells, in which *SLC7A11* is responsible for the main transport activity with high specificity for cystine and glutamate 22, 55. System x_c_^-^ intracellular glutamate is used to exchange extracellular cystine 24, 25, and then cystine is synthesized into GSH, catalyzed by glutamate-cysteine ligase and GSH synthetase. GSH is a reducing cofactor of the membrane lipid repair enzyme GPX4. Thus, increasing the activity of *SLC7A11* increases the absorption of cystine 56, 57, which affects GSH synthesis, increasing GPX4 activity 58, 59. Consequently, the antioxidant capacity of the cells is enhanced, inhibiting ferroptosis. Erastin induces ferroptosis, inducing ferroptosis by inhibiting the cysteine/glutamate reverse transporter system 60. Erastin also inhibits *SLC7A11*, providing a reasonable way to study the role of *AhR* in ferroptosis in NSCLC. In this study, Erastin was mainly used to inhibit *SLC7A11* and induce ferroptosis.In addition, the limitation of this experiment is that a complete pathway has not been found.

In conclusion, we demonstrated that *AhR* inhibits iron-dependent cell death (i.e., ferroptosis) in human non-small cell lung cancer cells by binding to the promoter region of *SLC7A11*, a transcription factor. Thus, it promotes *SLC7A11* transcription, influencing tumor cell ferroptosis. Specifically, *AhR* upregulated *SLC7A11* expression. Moreover, high *SLC7A11* levels increased cystine absorption, promoted GSH synthesis, increased GPX4 activity, enhanced the antioxidant capacity of lung cancer cells, inhibited lung cancer cell ferroptosis, and promoted cancer development. These findings highlight the indispensable role of AhR in tumor development via ferroptosis regulation, which may be a new treatment strategy for NSCLC.
